# First-line therapy with gemcitabine and paclitaxel in locally, recurrent or metastatic breast cancer: A phase II study

**DOI:** 10.1186/1471-2407-5-151

**Published:** 2005-11-29

**Authors:** Djelila Allouache, Sulochana R Gawande, Michele Tubiana-Hulin, Nicole Tubiana-Mathieu, Sophie Piperno-Neumann, Fawzia Mefti, Laurence Bozec, Jean-Yves Genot

**Affiliations:** 1 Centre F. Baclesse, Lion sur mer, 14076 CAEN Cedex 05, France; 2 Eli Lilly and Company, Lilly Corporate, Indianapolis, IN 46285, USA; 3 Ctr René Huguenin, 35 rue Dailly, 92210, Saint-Cloud, France; 4 Hopital Dupuytren, 2 avenue Martin Luther King, 87042, Limoges, France; 5 Hospital Curie, 26 rue Ulm, 75005, Paris, France; 6 Lilly France, 13 rue Pagès 92158 Suresnes Cedex, France

## Abstract

**Background:**

This phase II study evaluated the efficacy and safety of gemcitabine (G) plus paclitaxel (T) as first-line therapy in recurrent or metastatic breast cancer.

**Methods:**

Patients with locally, recurrent or metastatic breast cancer and no prior chemotherapy for metastatic disease received G 1200 mg/m^2 ^on days 1 and 8, and T 175 mg/m^2 ^on day 1 (before G) every 21 days for a maximum of 10 cycles.

**Results:**

Forty patients, 39 metastatic breast cancer and 1 locally-advanced disease, were enrolled. Their median age was 61.5 years, and 85% had a World Health Organization performance status (PS) of 0 or 1. Poor prognostic factors at baseline included visceral involvement (87.5%) and ≥2 metastatic sites (70%). Also, 27 (67.5%) patients had prior adjuvant chemotherapy, 25 of which had prior anthracyclines. A total of 220 cycles (median 6; range, 1–10) were administered. Of the 40 enrolled patients, 2 had complete response and 12 partial response, for an overall response rate of 35.0% for intent-to-treat population. Among 35 patients evaluable for efficacy the response rate was 40%. Additional 14 patients had stable disease, and 7 had progressive disease. The median duration of response was 12 months; median time to progression, 7.2 months; median survival, 25.7 months. Common grade 3/4 toxicities were neutropenia in 17 (42.5%) patients each, grade 3 leukopenia in 19 (47.5%), and grade 3 alopecia in 30 (75.0%) patients; 1 (2.5%) patient had grade 4 thrombocytopenia.

**Conclusion:**

GT exhibited encouraging activity and tolerable toxicity as first-line therapy in metastatic breast cancer. Phase III trials for further evaluation are ongoing.

## Background

In most developed countries, breast cancer is second only to lung cancer as the most common cause of cancer-related death in women, [1] and thus represents a serious health-care problem. Systemic therapy for patients with metastatic breast cancer (MBC) consists of hormonal therapy and cytotoxic chemotherapy. Anthracyclines such as doxorubicin and epirubicin can yield response rates of around 20% to 40% in MBC patients when used as single agents, and up to 60% when given as part of combination regimens [2]. However, the efficacy achieved with anthracyclines comes at the cost of high toxicity. New cytotoxic drugs with high activity, such as taxanes (paclitaxel and docetaxel), vinorelbine, gemcitabine, and capecitabine (all of which were introduced in the 1990s), have raised the hopes of patients with MBC to experience higher efficacy with tolerable toxicity. Recent studies suggest that combination chemotherapy may be more effective than single-agent therapy [3,4].

Paclitaxel is a mitotic spindle poison that promotes microtubular aggregation and interferes with essential cellular functions such as mitosis, cell transport, and cell motility [5,6]. It has shown remarkable activity in both chemonaive and anthracycline-resistant patients with MBC. Single-agent paclitaxel produced response rates of 32% to 62% in MBC patients not pretreated with chemotherapy, and from 6% to 48% in those who relapsed after treatment with anthracyclines [7-12].

Gemcitabine (difluorodeoxycytidine), an analog of cytosine arabinoside (ara-C), is a pyrimidine antimetabolite [13] that acts as a competitive substrate for incorporation into DNA where it brings about chain termination. It has undergone considerable testing for various malignancies and has exhibited activity in many solid tumors, including advanced or MBC. In MBC, single-agent gemcitabine has yielded response rates of up to 37% in chemonaive patients, [14-16] and 26% in those pretreated with anthracyclines [16-18]. Median progression-free survival with gemcitabine monotherapy was in the range of 2 to 6 months [14-20].

Preclinical studies of gemcitabine and paclitaxel have suggested that mechanisms of resistance affecting one drug may not have a significant effect on the other [21,22]. In pharmacokinetic studies, paclitaxel increased accumulation of dFdCTP, which might enhance the antitumor activity of gemcitabine in clinical studies [23]. The administration of paclitaxel prior to gemcitabine indicated an additive effect and was considered a better choice than a reverse sequence [24]. Study of this combination in treatment of ovarian cancer has also indicated that paclitaxel before gemcitabine is a less toxic sequence[25].

In summary, gemcitabine and paclitaxel are 2 agents with unique mechanisms of action, noncross-resistance, and the potential for synergistic antitumor activity. They are both active as single agents in the treatment of patients with MBC, and when used together, the treatment was well tolerated with promising activity [26-28]. A 3-week schedule of gemcitabine/paclitaxel (with gemcitabine administered on days 1 and 8, and paclitaxel on day 1) was tolerated better than a 4-week schedule [29,30].

Based on this information, we initiated a phase II, open-label, multicenter, nonrandomized study of gemcitabine in combination with paclitaxel given as first-line therapy in patients with recurrent or MBC. The primary objectives were to evaluate the response rate and duration of response. The secondary objectives were to assess safety, and to determine the time to disease progression and overall survival of this combination.

## Methods

### Eligibility criteria

Patients with unresectable, locally recurrent, or metastatic breast cancer that was not amenable to surgery or radiation of curative intent were enrolled. The qualifying patients were required to have histologically or cytologically confirmed breast carcinoma, with bidimensionally measurable lesions at least 1 cm × 1 cm (or 2 cm × 2 cm by physical examination). Prior chemotherapy was not allowed unless patients had local or metastatic relapse more than 12 months after the end of prior adjuvant or neoadjuvant chemotherapy. Patients could not have received previous therapy with gemcitabine or a taxane. Patients aged 18 to 75 years were further required to have a World Health Organization (WHO) performance status of 0 to 2, and an estimated life expectancy of at least 12 weeks. It was also necessary for patients to have adequate bone marrow function (absolute granulocyte count [AGC] ≥1.5 × 10^9^/L, platelet count ≥100 × 10^9^/L, and hemoglobin ≥90 g/L), adequate liver function (bilirubin ≤1.5 times the upper limit of normal [ULN], alanine transaminase [ALT] and aspartate transaminase [AST] ≤3 times ULN, or up to 5 times the ULN in patients with known metastatic liver disease), and adequate renal function (creatinine ≤2.5 times above the ULN). Prior radiation was permitted only if measurable disease was outside a previously irradiated area, if radiotherapy was not given to more than 50% of bone marrow volume, and if it was terminated at least 4 weeks prior to enrollment. Prior bone marrow transplantation, as adjuvant therapy, was allowed, and antitumoral hormonal treatment had to be terminated before enrollment. Patients with inflammatory breast cancer without evidence of metastatic disease, as well as patients who were pregnant, who had a neurological disorder WHO grade ≥2, or serious concomitant systemic disorders incompatible with the study could not participate. Patients were also excluded for active cardiac disease not controlled by therapy and/or myocardial infarction within the previous 6 months. Additional exclusion criteria included active infection, presence of severe psychiatric disease, or second malignancy (except *in situ *carcinoma of the cervix or adequately treated basal cell carcinoma of the skin).

All the patients who presented and met eligibility criteria were entered into the study. Physicians obtained signed informed consent from all patients prior to administering treatment. The study was conducted per the guidelines of good clinical practice and the Declaration of Helsinki.

### Treatment plan

Paclitaxel 175 mg/m^2 ^was administered intravenously (iv), before gemcitabine, on day 1 over a period of 3 hours. Gemcitabine 1200 mg/m^2 ^was given iv on days 1 and 8 over a period of 30 to 60 minutes (ideally 30 minutes), followed by a 1-week rest period. Each 21-day (3-week) period defined a cycle of therapy. Multiple injections of both drugs were administered for a total of at least 2 cycles unless it was clearly not in the patient's best interest to continue. Treatment was stopped in case of intolerable toxicity or disease progression. Additional cycles, up to a maximum of 10, could be administered in patients exhibiting a complete response (CR) or partial response (PR). All patients were premedicated with dexamethasone and chlorpheniramine, or clemastine or ranitidine or cimetidine prior to paclitaxel administration to prevent severe hypersensitivity reactions.

Dose adjustments during treatment (day 8) were made based on weekly AGC and platelet counts performed within 24 hours prior to the start of therapy and clinical assessment of nonhematologic toxicities. For an AGC <1.0 (×10^9^/L) and/or a platelet count <75 (×10^9^/L) or a WHO nonhematologic toxicity grade 3 (except nausea/vomiting and alopecia) or grade 4, doses of gemcitabine were held. A dose missed for any reason was not given at a later time.

The day-1 dose of each subsequent cycle depended on the toxicity seen in the previous cycle. The treatment was delayed until the AGC returned to 1.5 and the platelet count to 100. Otherwise, full doses of both drugs were given, except in patients with WHO grade 4 granulocytopenia lasting for more than 1 week, or grade 4 neutropenia associated with fever ≥38.5°C, or grade 4 thrombocytopenia. In these circumstances, after recovery, the day 1 and 8 doses of both drugs were given at 75% of the dose given on day 1 of the last cycle. The observed nonhematologic toxicities (except alopecia and vomiting) had to return to WHO grade 0 to 1, or baseline conditions, before resuming injections of both drugs. Doses in subsequent cycles were reduced to 75% or held for any grade 3 nonhematologic toxicity (except nausea/vomiting and alopecia), and were reduced to 50% or held for any grade 4 nonhematologic toxicity. Patients were withdrawn from the study after 3 weeks of treatment delay due to any toxicity.

### Baseline and treatment assessments

Assessments performed at baseline and throughout the study included history and physical examination (including weight and height), WHO performance status, and tumor measurement of palpable or visual lesions. Radiological tests of computed tomography (CT) scan, magnetic resonance imaging (MRI), or nuclear medicine scan were used, if necessary, for tumor measurement of lesions not evaluable by other imaging modalities. Chest x-rays were used in patients with chest metastasis. Full blood count (with differential and platelet counts), blood chemistries, electrocardiogram, and vital signs were done for all patients before and at regular intervals during the study. Additionally, coagulation studies were assessed as appropriate and the number of units required for transfusions every 4 weeks.

All patients who received at least 2 cycles of therapy and 1 radiologic evaluation were eligible for the efficacy analysis which was done based on WHO criteria. Complete response (CR) was defined as the disappearance of all known disease, and PR was defined as at least a 50% decrease in the total tumor size of the lesions, both determined by two observations not less than 4 weeks apart, and without appearance of new lesions. Stable disease (SD) was documented if a 50% decrease in total tumor size could not be established, nor a 25% increase in the size of one or more measurable lesions demonstrated. Progressive disease (PD) was defined as an increase ≥25% in measurable or evaluable tumor size and/or the appearance of new tumor sites. The safety analysis was performed on data from all patients who received at least 1 dose of the study drugs. The same assessment method used to determine the disease status at baseline was used consistently throughout the study for efficacy evaluation. Patients were clinically evaluated for response at the start of each cycle. Every 3 cycles during therapy, then every 3 months until disease progression, patients were assessed by radiologic imaging studies and chest radiography. Survival was measured from time of first-dose administration until the date of death. Time to disease progression was calculated from the administration of the first dose until the date of progression. Overall duration of response was measured from the first day of treatment to the date of the first observation of progressive disease. Toxicity ratings, based on WHO criteria, were assessed before the beginning of each next cycle.

### Data analysis

The response rate for this study was anticipated to be in the region of 40%. If 35 patients qualified for the efficacy analysis and 14 responses (40%) were observed, then the 95% confidence interval (CI) would be 26%–54%. The evaluability criteria for efficacy analysis were predefined in the protocol as; any patient who received at least 2 cycles of therapy and one radiological evaluation. It was anticipated that approximately 10% of the patients recruited might not qualify for the efficacy analysis. Consequently, this study intended to recruit 39 patients.

For the primary analysis, a 95% CI for the true response rate was calculated using the normal approximation to the binomial distribution. Estimates of duration of response and time to disease progression were calculated using the Kaplan-Meier method, with medians and quartiles derived from these estimates.

## Results

### Patient characteristics

From October 1999 to December 2001, a total of 40 female patients were entered, and all were enrolled (received study treatment). These patients had a median age of 61.5 years (range, 30–76 years), and the majority (85%) had a WHO performance status of 0 or 1. The baseline patient and disease characteristics are summarized in Table 1. This patient population presented with multiple poor prognostic factors. Overall, 39 (97.5%) patients presented with metastatic disease, and 35 (87.5%) had visceral involvement. Eight (20.0%) patients reported both liver and lung metastases at baseline, and 28 (70.0%) patients had at least 2 sites of metastatic disease at baseline. Three patients had 4 or more tumor burden sites, including 1 patient with 6 sites of metastatic disease. Of the 35 patients with known hormone receptor status, 12 (34.3%) were negative for estrogen/progesterone receptors (ER/PR). In addition, 27 (67.5%) patients had received previous adjuvant chemotherapy, 25 of whom had prior anthracyclines. A total of 24 (60.0%) patients had received both adjuvant chemotherapy and hormonal therapy.

### Response and time-to-event measures

Among the 40 patients enrolled, there were 2 CR and 12 PR. Based on an Intent-to-treat analysis, the overall response rate was 35.0%. An additional 14 (35.0%) patients had stable disease, and 7 patients (17.5%) had a best response of progressive disease. Of the remaining 5 patients, 4 did not undergo further evaluation of their lesions after baseline, and 1 patient did not have histologically or cytologically proven breast carcinoma. As per the predefined criteria in the protocol these 5 patients were not eligible for efficacy analysis.

According to per-protocol efficacy analysis, 2 CR and 12 PR, gave an overall response rate of 40.0% (14/35) (95% CI, 23.8%–56.2%). The response rate was lower among 22 patients who had prior anthracycline treatment compared to the remaining 13 with no anthracycline exposure (27.3% vs 61.5%) (Table 2).

At the time of this analysis, the median follow-up time (the period from patient enrollment to last visit) was 13.7 months (range, 1.6–28.3 months). A total of 14.3% (n = 5) of patients were censored for the estimate of duration of response because they were alive and progression-free, or lost to follow-up at the time of the analysis. The estimate of median duration of response was 12.0 months (95% CI, 10.0–15.0 months). The probability of duration of response lasting at least 9 and 12 months was estimated as 78.6% (95% CI, 57.1%–100%) and 47.1% (95% CI, 20.0%–74.3%), respectively. The estimate for median time to progressive disease was 7.2 months (95% CI, 4.6–10.0 months). The progression-free probability at 6 and 9 months was estimated as 62.9% (95% CI, 46.8%–78.9%) and 40.0% (95% CI, 23.8%–56.2%), respectively. A total of 11.4% (n = 4) of patients who were alive and had not progressed, or were lost to follow-up, were censored for this analysis. The most common sites of disease progression were liver and lung, which were the most commonly involved sites at baseline. Fourteen patients had died, and the remaining 60% (n = 21) of patients who were alive or lost to follow-up were censored for the survival analysis. The estimate of median survival was 25.7 months (95% CI, 14.7-xx months). The probability of surviving beyond 12 and 18 months was estimated as 74.0% (95% CI, 59.3%–88.6%) and 62.0% (95% CI, 44.5%–79.5%), respectively.

### Dose administration

All 40 patients received at least 1 cycle of therapy, for a total of 220 cycles. The median number of cycles given was 6 (range, 1–10 cycles). Three (7.5%) patients completed the study and received the maximum number of 10 treatment cycles. The most frequently cited reason for discontinuation from the study was progressive disease (14 patients; 35.0%), as indicated by radiologic and/or physical assessment. Five (12.5%) patients discontinued the study because of adverse events, and 1 patient died in cycle 2 due to progression.

Patients received mean doses of 681.0 mg/m^2 ^gemcitabine and 56.7 mg/m^2 ^paclitaxel per week, which were 85.1% and 97.2% of the planned weekly mean doses, respectively. There were 48 (10.9% of doses administered) dose omissions and 1 (0.2%) dose reduction of gemcitabine, and 4 (1.8%) omissions and 4 (1.8%) reductions of paclitaxel. Neutropenia was the most frequent cause of gemcitabine dose omission (35 of 48 doses omitted; 72.9%). All gemcitabine dose omissions due to neutropenia occurred on day 8, and the majority of dose omissions (22 of the 35 doses) occurred during the first 3 cycles. Paresthesia was the cause of 2 paclitaxel dose reductions and all 4 paclitaxel dose omissions. Overall, there were 34 cycle delays of administration of gemcitabine plus paclitaxel. The clinically relevant reasons for cycle delays included hepatitis in 1 patient (delay at cycles 2 and 3), pyrexia in 2 patients, neutropenia in 3 patients, and asthenia, edema, paresthesia, and pleural disorder in 1 patient each.

### Toxicity

All 40 patients entered into the study were evaluable for toxicity. The WHO grade 3 and 4 toxicities observed during this study were primarily hematologic (Table 3). These included grade 3 and 4 neutropenia in 17 (42.5%) patients each, grade 3 leukopenia in 19 (47.5%) patients, grade 3 anemia in 2 (5.0%) patients, and grade 3 and 4 thrombocytopenia in 1 (2.5%) patient each. Although 2 patients with grade 4 neutropenia had concurrent fever, neither patient was hospitalized for febrile neutropenia. The patient who reported grade 4 thrombocytopenia required a platelet transfusion. A total of 8 units of red blood cells were given; the recipients included both of the patients with grade 3 anemia.

No grade 4 nonhematologic toxicities were reported during the study. The most common grade 3 toxicity was alopecia in 30 (75.0%) patients. Three (7.5%) patients reported grade 3 peripheral neurotoxicity, which occurred during later cycles (cycles 4 through 8). Grade 2 neurotoxicity was experienced by 12 (30.0%) patients. There was only 1 report of a grade 2 pulmonary toxicity that was described as dyspnea. One patient, who was previously treated in the adjuvant setting with epirubicin, 5-FU, and cyclophosphamide combination therapy and radiotherapy, reported cardiac toxicity. The patient was hospitalized for severe dyspnea and was diagnosed with arrhythmia and tachycardia. The patient also had grade 3 infection described as severe sepsis. The patient discontinued the study due to cardiac rhythmic events.

There were 2 deaths, 1 of which occurred on-study and 1 during the 30-day follow-up period after administration of the last dose of study drug. Both of the deaths were considered related to disease progression. There were no deaths due to study drug toxicity.

## Discussion

The results of this phase II trial demonstrated that the combination of gemcitabine 1200 mg/m^2 ^on days 1 and 8, plus paclitaxel 175 mg/m^2 ^on day 1, administered in a 21-day cycle was effective in patients with unresectable, locally recurrent or MBC. In a first-line setting, this regimen resulted in a response rate of 40.0% (35.0% for Intent-to-treat population), with a complete response rate of 5.7%. The response rate in the patients previously exposed to anthracyclines was lower than in those not exposed to anthracyclines. The median duration of response was 12 months. In addition, the median time to progressive disease was 7.2 months, which was higher than that obtained with either single agent. Although the overall survival data are not fully mature, the median survival time of 25.7 months is also quite promising. This is a phase II non-randomized trial, and hence there are limitations in interpreting these results, however the efficacy results compare favorably with those seen with single-agent paclitaxel or single-agent gemcitabine in MBC,[11,14,31,32] and are also comparable with the results of this combination seen in other trials (see Table 4). It is important to note that these efficacy results were achieved in a patient population with multiple poor prognostic factors such as: visceral involvement, at least 2 sites of metastatic disease at baseline, negative hormone receptor status, and prior exposure to chemotherapy, including anthracyclines, in an adjuvant setting.

Since the initiation of this trial, the combination of gemcitabine and paclitaxel has been tested in different doses and schedules, as well as in various patient populations with different levels of exposure to prior chemotherapy. In a phase I dose-finding study,[33] fixed doses of gemcitabine (1000 mg/m^2^) on days 1 and 8 were administered with escalating doses of paclitaxel (range, 90–270 mg/m^2^) on day 1 of a 21-day cycle in patients with pretreated MBC or ovarian cancer. At the paclitaxel dose level of 270 mg/m^2^, the dose-limiting toxicities (DLT) of grade 4 neutropenia and thrombocytopenia were noted, but there were no unexpected toxicities. Among 30 evaluable patients with MBC, 4 CRs (13%) and 12 PRs (40%) were observed, for an overall response rate of 53%. The median duration of response was 7.2 months.

In another study, heavily pretreated MBC patients were treated with 2500 mg/m^2 ^gemcitabine and 135 mg/m^2 ^paclitaxel, both of which were given on days 1 and 15 [29]. The overall response rate achieved was 45%. The median time to progression was 7 months, and overall survival time was 11 months. However, 34% of the patients needed growth factors. In a similar trial, Vici et al [26] used 1500 mg/m^2 ^gemcitabine and 135 mg/m^2 ^paclitaxel on days 1 and 15 every 4 weeks in heavily pretreated advanced breast cancer patients who had received 1 to 4 cycles of prior chemotherapy. Although the doses were lower than those used in the Sanchez-Rovira trial, patients were supported with G-CSF injections. In the preliminary results, the overall response rate in 20 evaluable patients was 45% with a median time to progression of 8 months.

Murad et al [30] evaluated gemcitabine plus paclitaxel in heavily pretreated MBC patients with history of 2 or 3 relapses following treatment with anthracycline-containing regimens. The initial schedule was gemcitabine (1000 mg/m^2^) on days 1, 8, and 15 plus paclitaxel (175 mg/m^2^) on day 1, given every 4 weeks. However, due to occurrence of unacceptable toxicity (thrombocytopenia) in the first 5 patients, the schedule was modified to every 3 weeks with gemcitabine given on days 1 and 8. The modified regimen was well tolerated, with a significantly lower incidence of grade 3 or 4 thrombocytopenia (18.5% in the day-28 schedule vs 5.4% in the day-21 schedule), and resulted in an overall response rate of 55%, with 17% complete responses. The median response duration was 8 months, and the median overall survival was 12 months.

The combination of gemcitabine and paclitaxel was also evaluated as first-line treatment of advanced or MBC. Forty-three chemonaive patients with histologically confirmed metastatic breast carcinoma received paclitaxel 150 mg/m^2 ^followed by gemcitabine 2500 mg/m^2^, both on day 1 of a 2-week cyclem [27]. Among the 38 evaluable patients, the overall response rate was 68%, with moderate neutropenia seen in 32% of patients.

Delfino et al [28] assessed the efficacy and toxicity of the gemcitabine/paclitaxel combination in a first-line setting using a 3-week schedule. Chemonaive patients with advanced or metastatic breast cancer were given paclitaxel (175 mg/m^2^) on day 1 and gemcitabine (1200 mg/m^2^) on days 1 and 8 every 3 weeks. The overall response rate was 67%, with 22% complete responders. The median time to tumor progression was 11 months, and the median duration of response was 18 months.

The results of these studies (Table 4), along with those of our current study, clearly establish the efficacy of the gemcitabine and paclitaxel combination in chemonaive and pretreated MBC patients. At present, anthracycline-based combinations are the mainstay of chemotherapy in the early treatment of breast cancer, but their effectiveness decreases in later treatments in the metastatic setting. The cardiotoxicity associated with anthracyclines also limits the total amount of the drug that can be used in a patient. Thus, there is a strong need to develop newer treatment regimens that are not cross-resistant with anthracyclines yet have antitumor activity in MBC. Gemcitabine and paclitaxel are known to possess considerable cytotoxic activity individually with minimally overlapping toxicity profiles. Both drugs act on different cellular targets with indications of noncross-resistance to each other. In pharmacokinetic studies, gemcitabine and paclitaxel did not interfere with each other, and *in vitro *studies did not demonstrate any synergism between these 2 drugs [23,34]. However, increased accumulation of dFdCTP by paclitaxel might augment the antitumor activity of gemcitabine in clinical studies[23]. Based on this information, it is not unreasonable to expect that the addition of paclitaxel to gemcitabine may produce additional efficacy that is superior to either drug given alone. To explore this possibility further, a phase randomized III trial comparing gemcitabine plus paclitaxel versus paclitaxel alone is currently under way. The efficacy and toxicity results of this study will offer significant insight into the clinical implications of combining these agents in patients with advanced or metastatic breast cancer.

## Conclusion

In conclusion, gemcitabine in combination with paclitaxel has demonstrated notable activity along with an acceptable and tolerable safety profile. Further evaluation of this regimen is warranted in the treatment of metastatic breast cancer.

## Competing interests

Laurence Bozec was employed by Eli Lilly France as a clinical research physician at the time of the writing of this manuscript. Sulochana Gawande is currently employed by Eli Lilly and Company.

The remaining authors, Djelila Allouache, Michele Tubiana-Hulin, Nicole Tubiana-Mathieu, Sophie Piperno-Neumann, Fawzia Mefti, and Jean-Yves Genot, have no competing interests.

## Authors' contributions

Djelila Allouache conducted patient trial, participated in patient enrollment and manuscript review.

Sulochana R. Gawande helped in data analysis, data interpretation and writing the manuscript.

Michele Tubiana-Hulin conducted patient trial, participated in patient enrollment and manuscript review.

Nicole Tubiana-Mathieu conducted patient trial, participated in patient enrollment and manuscript review.

Sophie Piperno-Neumann conducted patient trial, participated in patient enrollment and manuscript review.

Fawzia Mefti conducted patient trial, participated in patient enrollment and manuscript review.

Laurence Bozec helped in conducting the trial, data collection and writing the manuscript.

Jean-Yves Genot conducted patient trial, participated in patient enrollment and manuscript review.

## Pre-publication history

The pre-publication history for this paper can be accessed here:



## Figures and Tables

**Table 1:** Patient characteristics (N = 40)

Patients entered and enrolled	40
Median age, years (range)	61.5 (30–76)
WHO performance status, n (%)	
0	18 (45.0)
1	16 (40.0)
2	6 (15.0)
Histology, n (%)	
Breast	1 (2.5)
Ductal breast	31 (77.5)
Lobular breast	8 (20.0)
Number of metastatic sites, n (%)	
1	12 (30.0)
2	21 (52.5)
3	4 (10.0)
≥4	3 (7.5)
Metastatic sites of disease^a^, n (%)	
Visceral	35 (87.5)
Liver	26 (65.0)
Lung (including pleura)	17 (42.5)
Nonvisceral only	5 (12.5)
Menopausal status, n (%)	
Pre-menopausal	20 (50.0)
Menopausal	8 (20.0)
Post-menopausal	10 (25.0)
Unknown	2 (5.0)
Estrogen/progesterone receptors, n (%)	
Positive	23 (57.5)
Negative	12 (30.0)
Unknown	5 (12.5)

^a^Patients may be counted in more than one category.

**Table 2:** Summary of best tumor response according to prior anthracycline exposure

	**No. of Pts**	**No. of Responders**	**Percent of Pts**	**95% CI**
Evaluable patients	35	14	40.0%	(23.8%–56.2%)
Prior anthracycline exposure				
Yes	22	6	27.3%	(8.7%–45.9%)
No	13	8	61.5%	(35.1%–88.0%)

CI = confidence interval.

**Table 3:** Summary of maximum WHO toxicity grades (N = 40)

	**Grade 3**		**Grade 4**	
	
**Toxicity**	**n**	**(%)**	**n**	**(%)**
*Hematologic*				
Anemia	2	5.0	0	0
Leukopenia	19	47.5	0	0
Neutropenia^a^	17	42.5	17	42.5
Thrombocytopenia	1	2.5	1	2.5

*Non-hematologic*				
Cardiac function	1	2.5	0	0
Alopecia	30	75.0	0	0
Infection	1	2.5	0	0
Nausea/vomiting	1	2.5	0	0
Peripheral neurotoxicity	3	7.5	0	0

^a^Segmented neutrophils have been converted to WHO scores using granulocyte count criteria.

**Table 4:** Phase II studies of gemcitabine/paclitaxel in patients with advanced or metastatic breast cancer

**Author**	**Regimen**	**No. of Pts**	**ORR % (CR %)**	**Median TTP (mos)**	**Comments**
Vici et al^26^	G 1500 mg/m^2 ^d1, 15 + T150 mg/m^2 ^d1, 15 q4wks	20	45% (10%)	8	Heavily pretreated pts; supported with G-CSF
Colomer et al^27^	G 2500 mg/m^2 ^d1 + T150 mg/m^2 ^d1 q2wks	43	68% (21%)	9	Previously treated pts; hematologic toxicity
Delfino et al^28^	G 1200 mg/m^2 ^d1, 8 + T 175 mg/m^2 ^d1 q3wks	45	67% (22%)	11	Chemonaïve pts; mild hematologic toxicity; manageable toxicity
Sanchez-Rovira et al^29^	G 2500 mg/m^2 ^d1, 15 + T 135 mg/m^2 ^d1, 15 q4wks	44	45% (7%)	7	Previously treated pts; hematologic and neurologic toxicities
Murad et al^30^	G 1000 mg/m^2 ^d1, 8 + T 175 mg/m^2 ^d1 q3wks	29	55% (17%)	8	Heavily pretreated pts; 28-day reduced to a 21-day schedule due to hematologic toxicity
Genot et al (current study)	G 1200 mg/m^2 ^d1, 8 T 175 mg/m^2 ^d1 q3wks	40	40% (5.7%)	7.2	No prior treatment for metastatic disease, neutropenia prominent, other toxicities mild

ORR = overall response rate; CR = complete response; TTP = time to progression; mos = months; G = gemcitabine; T = paclitaxel.
